# Effectiveness of extracorporeal blood purification (hemoadsorption) in patients with severe coronavirus disease 2019 (COVID-19)

**DOI:** 10.1186/s12882-020-02020-3

**Published:** 2020-08-20

**Authors:** Masoumeh Asgharpour, Hamed Mehdinezhad, Masoumeh Bayani, Mahmoud Sadeghi Haddad Zavareh, Seyed Hossein Hamidi, Roghayeh Akbari, Reza Ghadimi, Ali Bijani, Simin Mouodi

**Affiliations:** 1 Department of Internal Medicine, Rouhani Hospital, Babol University of Medical Sciences, Babol, Iran; 2 Infectious Diseases and Tropical Medicine Research Center, Health Research Institute, Babol University of Medical Sciences, Babol, Iran; 3 Department of Anesthesiology, School of Medicine, Babol University of Medical Sciences, Babol, Iran; 4 Department of Internal Medicine, Babol University of Medical Sciences, Babol, Iran; 5 Social Determinants of Health Research Center, Health Research Institute, Babol University of Medical Sciences, Ganjafrooz Street, Babol, Iran

**Keywords:** Coronavirus infection, Extracorporeal dialysis, Critical illness

## Abstract

**Background:**

Extracorporeal blood purification has been proposed as one of the therapeutic approaches in patients with coronavirus infection, because of its beneficial impact on elimination of inflammatory cytokines.

**Methods:**

This controlled trial has been conducted on critically ill COVID-19 patients admitted in the state hospital affiliated to Babol University of Medical Sciences, Iran who received different antiviral and antibacterial drugs, and different modalities of respiratory treatments and did not have positive clinical improvement. No randomization and blindness was considered. All of the participants underwent three sessions of resin-directed hemoperfusion using continuous renal replacement therapy with a mode of continuous venovenous hemofiltration (CVVH).

**Results:**

Five men and five women with a mean age of 57.30 ± 18.07 years have been enrolled in the study; and six of them have improved after the intervention. Peripheral capillary oxygen saturation (SpO2) changed after each session. Mean SpO2 before the three sessions of hemoperfusion was 89.60% ± 3.94% and increased to 92.13% ± 3.28% after them (*p* < 0.001). Serum IL-6 showed a reduction from 139.70 ± 105.62 to 72.06 ± 65.87 pg/mL (*p* = 0.073); and c-reactive protein decreased from 136.25 ± 84.39 to 78.25 ± 38.67 mg/L (*P* = 0.016).

**Conclusions:**

Extracorporeal hemoadsorption could improve the general condition in most of recruited patients with severe coronavirus disease; however, large prospective multicenter trials in carefully selected patients are needed to definitely evaluate the efficacy of hemoperfusion in COVID-19 patients.

**Trial registration:**

The research protocol has been registered in the website of Iranian Registry of Clinical Trials with the reference number IRCT20150704023055N2.

## Background

Corona viruses are a large family of viruses that can lead to a wide range of diseases; from a simple common cold to severe health problems that have attracted much attention in the world, such as the Middle East Respiratory Syndrome (MERS-CoV) and Severe Acute Respiratory Syndrome (SARS) [1]. Corona virus disease 2019 (COVID-19) is a new strain of this viral family that was first introduced in China in 2019 and has never been seen in human populations before [2, 3]. According to the World Health Organization, a total of 1,773,084 confirmed cases and 111,652 deaths have been reported worldwide until April 132,020; of these, 99,713 confirmed and 5107 deaths were from the Eastern Mediterranean Region [4].

Different clinical features have been reported for COVID-19 so far, from an asymptomatic form to a severe disease leading to respiratory failure requiring intensive-care treatment and mechanical ventilation, multiple organ involvement and even multiple organ failure [5]. Common manifestations of confirmed cases have been listed as fever, fatigue, dry cough, nasal congestion, shortness of breath, myalgia and arthralgia with laboratory findings such as lymphopenia, high plasma levels of c-reactive protein, and elevated lactate dehydrogenase; 7–10% of patients may progress to critical cases; and about 1–2% of cases will result in death; of course, the mortality rate of patients varies according to their geographical location [3].

Similar to other outbreaks caused by new viral agents, a definite approved treatment has not yet been introduced, and treatment protocols presented in scientific evidence are all symptomatic and supportive [3, 5]. Patients who develop acute respiratory distress syndrome are more likely to require ICU care and die, and the cytokine storm has been found to be associated with the severity of the disease [6]. It has been demonstrated that at the beginning of sepsis process, the overshoot of multiple pro-inflammatory mediators is frequently observed, and patient mortality will be much higher when the serum level of pro-inflammatory and anti-inflammatory cytokines is considerable [6, 7]. Blocking the overshoot of these inflammatory mediators can stop the sepsis process and improve patient outcomes [7]. One of the treatment approaches that can be taken to reduce these cytokines is extracorporeal blood purification, also referred to as hemoperfusion [8–11]. Hemoperfusion is an extracorporeal technique involving the passage of blood through a cartridge where solutes are removed by direct binding to the sorbent material. Hemoperfusion acts by adsorption mechanism, related to different cartridges which have been provided in its structure. It differs from hemodialysis because hemodialysis acts by diffusion mechanism. In continuous venovenous hemofiltration (CVVH) mode of hemoperfusion, the mechanism of convection is added, and no diffusion is occurred [12]. The effectiveness of hemoperfusion on serum level of IL-6, IL-8, IL-1β, and tumor necrosis factor has been demonstrated in some previous studies [13]. Four extracorporeal therapies have been introduced for cytokine removal in patients with COVID-19: continuous renal replacement therapy (CRRT) with hollow fibre filters with adsorptive properties; direct hemoperfusion using a neutro-macroporous sorbent; plasma adsorption on a resin after plasma separation from the whole blood; and high-dose CRRT with medium or high cut-off membranes [10].

Considering the association of increased cytokines release with severity of COVID-19 disease, and the effect of hemoperfusion on removal of these cytokines [7, 13], this study conducted to determine the effectiveness of hemoperfusion in patients with severe coronavirus disease 2019.

## Methods

This controlled trial has been performed on adult patients (aged 18 years and over) with severe COVID-19 disease admitted in the state hospital affiliated to Babol University of Medical sciences, Iran; since 2020/03/10 to 2020/03/23. Patients who had clinical manifestations of COVID-19 in addition to positive radiographic (lung CT-scan) findings or laboratory confirmation by testing of the oropharyngeal specimen, using real-time polymerase chain reaction were included, if they had one of these criteria: individuals who had partial pressure of oxygen in alveoli (PaO2) less than 60 mmHg, even after different methods of oxygen-therapy; or peripheral capillary oxygen saturation (SpO2) less than 88% with no clinical improvement despite 48 h of non-invasive respiratory therapy. The individuals recruited in the research when the informed consent was obtained. Exclusion criteria were plasma platelet count less than 30,000 per microliter, and multiple organ dysfunction.

The patients underwent extracorporeal blood purification on three sessions [10], using continuous renal replacement therapy (CRRT) machine manufactured by the B. Braun Company, Germany, with a mode of continuous venovenous hemofiltration. Jugular temporary catheter (Bard trademark) was placed by a vascular surgeon. Heparin has been injected as an anticoagulant agent throughout the CRRT by the arterial line depending on the patient’s coagulation status [14]. The blood flow rate (QB) was 200–250 mL/minute, the hemofiltration fluid flow rate (QD) 2Lit/hour, and the effluent volume was 2lit/hour. During the CRRT procedure, the fluid outflow from the patient was adjusted as the same amount of the fluid input.

Each session conducted in 14–18 h per day; the first 4–6 h with CRRT plus hemoperfusion; and the last 10–12 h with CRRT alone. We used resin-directed hemoadsorption cartridges (HA-280 and HA-230) manufactured by the Jafron Biomedical Company, China. The second course of hemoperfusion was performed 24–48 h after the first and the third session 24–48 h after the second time. Since the patients with severe coronavirus disease have been enrolled in the study, neither control group nor randomisation was considered. Anti-inflammatory and antiviral medications have been controlled and patients who had nearly similar treatment conditions were included in the study.

Age, gender, duration of hospital and ICU admission due to COVID-19, comorbid disorders, medications received for the recent disease; vital signs including body temperature, pulse rate, blood pressure, and respiratory rate; laboratory tests including plasma white blood cell count, hemoglobin, plasma platelet count, serum creatinine, blood urea nitrogen, serum c-reactive protein and lactate dehydrogenase, hepatobiliary function tests (AST, ALT, and total and direct bilirubin); and SpO2 have been recorded in the research data sheet. In addition to, the mode of oxygen therapy has been recorded before and after each session of the hemoperfusion.

Primary outcome was improving the general condition based on the patient’s assessment 1 week after the third session of hemoperfusion compared to the initial clinical condition before the first hemoperfusion. The patient was considered as improved if he did not need to receive any intensive respiratory treatment, based on the status of peripheral capillary oxygen saturation. Furthermore, the serum level of interleukin-6 (IL-6) has been measured as the secondary outcome of the study [15].

Required clinical criteria used to determine the patient’s readiness for weaning from mechanical ventilation included: improvement of the cause of the respiratory failure; hemodynamic stability; PaO2/FiO2 ≥ 150 or SpO2 ≥ 90% on FiO2 ≤ 40% and positive end-respiratory pressure ≤ 5 cmH2O; PH > 7.25 and able to initiate an inspiratory effort [16].

Data analysis was performed using SPSS-18 software package. Paired t-test was used to assess SpO2 in each patient, before and after the intervention; and t-test was used to analyze the examined demographic variables. To compare inflammatory variables which did not have normal distributions, Wilcoxon signed ranks test and Mann-Whitney test were used.

This clinical trial adheres to CONSORT guidelines. A completed CONSORT checklist has been provided as Appendix 1.

This research has been approved by the Ethics Committee of Babol University of Medical Sciences with the approval code IR.MUBABOL.HRI.REC.1399.038. Also, the research protocol has been registered in the website of Iranian Registry of Clinical Trials with the reference number IRCT20150704023055N2.

## Results

**Fig. 1:**
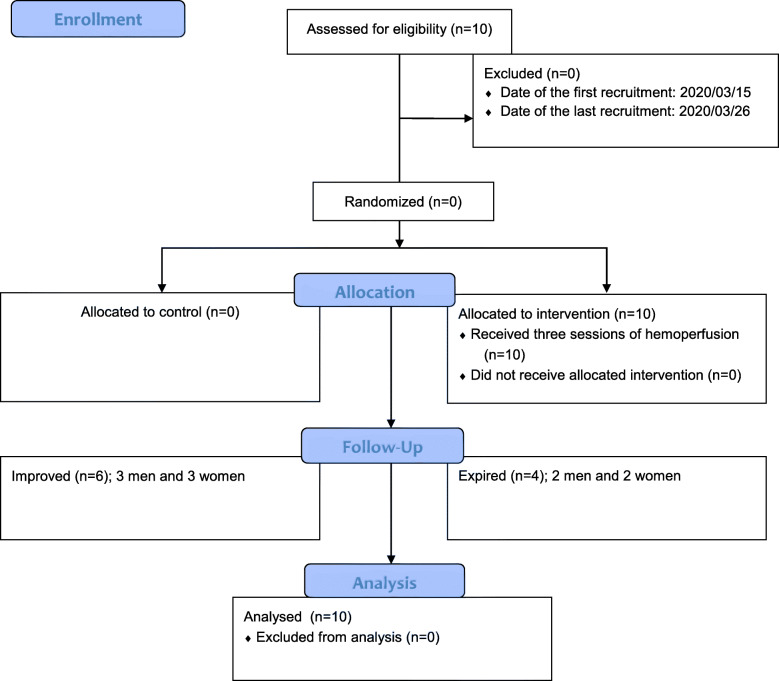
Flow diagram of the participants

Ten critically ill COVID-19 patients with a mean age of 57.30 ± 18.07 (a range from 26 to 83) years have been enrolled in the study. The flow diagram of the participants has been presented in Fig. 1. Five of them (50.0%) were male and five (50.0%) were female. The hemoperfusion treatment was started in patients 1–8 (average 4.7) days after the hospitalization. In one patient the second course of hemoperfusion was conducted 4 days after the first, because of some difficulties in providing the hemoperfusion cartridges; and others underwent the intervention according to the planned program.

Baseline characteristics of the participants have been presented in Table 1; laboratory measures before the first and the third session of hemoperfusion, and the treatment outcome have been summarized in Table 2. Six of the ten enrolled patients have been improved. The measured IL-6 mean serum level before the intervention was 139.70 ± 105.62 pg/mL and decreased to 72.06 ± 65.87 after the third session of hemoperfusion (*p* = 0.073). Mean serum level of c-reactive protein before and after the intervention was 136.25 ± 84.39 and 78.25 ± 38.67 mg/L, respectively (*P* = 0.016). Laboratory measures of the two expired and improved groups have been compared in Table 3. The percentage of plasma lymphocytes had a statistically significant difference between the two groups, the patients who have not been improved showed a lower plasma lymphocytes count (*p* = 0.038).

**Table 1:** Baseline characteristics of the participants

Patient’s serial number	Date of symptom onset	Date of hospital admission	Date of ICU admission	Underlying disorders	Medications received before the onset of hemoperfusion	Vital signs before the first session of hemoperfusion				Date of hemoperfusion sessions		
						Body Temperature (°C)	Blood Pressure (mm/Hg)	Pulse Rate (per minute)	Respiratory Rate (per minute)	The 1st	The 2nd	The 3rd
1	2020-03-05	2020-03-10	2020-3-13	Single kidney and chronic kidney disease	Linezolid, Kaletra (Lopinavir 400 and Ritonavir 100), Hydroxychloroquine, Meropenem, Oseltamivir 75	36.2	131/70	67	24	2020-03-17	2020-03-19	2020-03-21
2	2020-03-05	2020-03-11	2020-03-14	Pneumoconiosis	Vancomycin, Linezolid, Kaletra (Lopinavir 400 and Ritonavir 100), Hydroxychloroquine, Oseltamivir 75	37.0	153/100	99	28	2020-03-17	2020-03-18	2020-03-20
3	2020-03-08	2020-03-11	2020-03-13	No	Vancomycin, Kaletra (Lopinavir 400 and Ritonavir 100), Hydroxychloroquine, Meropenem, Oseltamivir 75	38.0	100/60	100	25	2020-03-18	2020-03-19	2020-03-20
4	2020-03-10	2020-03-14	2020-03-15	No	Vancomycin, Linezolid, Kaletra (Lopinavir 400 and Ritonavir 100), Hydroxychloroquine, Meropenem, Oseltamivir 75	37.5	112/80	80	23	2020-03-16	2020-03-17	2020-03-18
5	2020-03-10	2020-03-14	2020-03-15	No	Vancomycin, Kaletra (Lopinavir 400 and Ritonavir 100), Azithromycin, Hydroxychloroquine, Meropenem, Oseltamivir 75	37.0	130/75	85	40	2020-03-15	2020-03-16	2020-03-18
6	2020-03-10	2020-03-15	2020-03-17	No	Vancomycin, Kaletra (Lopinavir 400 and Ritonavir 100), Hydroxychloroquine, Meropenem	37.7	143/88	93	22	2020-03-23	2020-03-24	2020-03-25
7	2020-03-10	2020-03-16	2020-03-19	Malignancy (Leukemia)	Vancomycin, Kaletra (Lopinavir 400 and Ritonavir 100), Hydroxychloroquine, Meropenem, Oseltamivir 75	36.8	123/96	108	30	2020-03-21	2020-03-22	2020-03-23
8	2020-03-10	2020-03-16	2020-03-19	Cardiovascular disorders, Hypertension, Coronary artery bypass graft surgery, Diabetes mellitus, Parkinson, Rheumatoid arthritis	Vancomycin, Kaletra (Lopinavir 400 and Ritonavir 100), Hydroxychloroquine, Meropenem, Oseltamivir 75	36.5	110/70	100	18	2020-03-20	2020-03-21	2020-03-22
9	2020-03-10	2020-03-17	–	Diabetes mellitus	Linezolid, Kaletra (Lopinavir 400 and Ritonavir 100), Meropenem	37.7	110/70	91	35	2020-03-21	2020-03-22	2020-03-23
10	2020-03-20	2020-03-23	2020-03-27	Cardiovascular disorders, Hypertension, Diabetes mellitus, Chronic kidney disease	Linezolid, Kaletra (Lopinavir 400 and Ritonavir 100), Hydroxychloroquine, Meropenem	38.5	100/57	57	26	2020-03-26	2020-03-30	2020-04-01

**Table 2:** Laboratory exams, respiratory measures and final outcome of the participants before the first and after the third session of hemoperfusion

Patient’s serial number	Laboratory exams before the first and after the third session of hemoperfusion																SpO2 before and after each session of hemoperfusion			Patient’s outcome
	WBC count	PMN (%)	Lymph (%)	HgB	PLT	CRP	LDH	BUN	Creatinine	Na	K	AST	ALT	Bill-Total	Bill-Direct	IL-6	The first session	The second session	The third session	
	Before	Before	Before	Before	Before	Before	Before	Before	Before	Before	Before	Before	Before	Before	Before	Before	Before	Before	Before	
	After	After	After	After	After	After	After	After	After	After	After	After	After	After	After	After	After	After	After	
1	14,600	92.1	4.8	8.8	345,000	178	1130	98	4.3	137	4.1	24	22	1.9	1.2	16	83	90	92	Expired
	21,400	91.3	4.9	6.4	75,000	220	1450	79	2.7	133	4.3	376	149	1.8	1.0	95	88	91	95	
2	9200	86.8	5.4	11.5	172,000	282	782	28	0.9	141	3.9	42	30	4.1	2.6	84	81	88	92	Improved
	10,300	77.2	6.4	9.8	235,000	150	574	60	2.8	137	4.7	40	23	3.8	2.4	88	86	91	96	
3	25,300	90.1	5.5	11.2	203,000	175	1650	15	0.6	132	3.6	43	28	1.8	0.8	351	89	93	93	Expired
	18,700	88.0	2.0	8.2	245,000	90	1495	20	0.6	143	3.6	39	27	5.6	4	88	88	90	12	
4	13,700	91.1	6.8	9.6	233,000	178	825	12	0.6	130	3.3	34	23	0.8	0.2	57	88	93	95	Improved
	13,300	88.4	7.6	9.0	351,000	13	690	19	0.7	135	3.1	32	30	1.4	1.1	9.4	90	94	97	
5	9900	92.1	5.6	13.8	235,000	84	727	12	1.1	133	3.9	94	57	1.2	0.4	97	88	90	97	Improved
	12,200	86.9	8.4	13.1	218,000	30	622	22	0.8	135	4.7	92	54	1.0	0.3	17	91	95	97	
6	7800	77.3	12.2	11.5	508,000	120	950	34	1.0	146	4.3	68	69	0.9	0.3	60	87	90	94	Improved
	6400	68.0	17.0	10.5	327,000	66	870	40	1.2	146	4.1	46	49	1.6	0.8	29.5	90	92	96	
7	3800	92.0	6.0	10.9	104,000	223	–	25	0.8	135	3.4	299	470	1.6	0.9	212	87	90	95	Improved
	6000	90.0	6.0	8.5	101,000	95	–	9	0.5	139	3.8	229	450	1.5	0.7	102	89	91	97	
8	12,200	72.0	1.6	12.4	162,000	69	–	61	1.6	131	4.1	58	22	0.4	0.2	215	82	86	89	Expired
	9200	68.0	2.5	11.1	155,000	42	–	59	1.4	132	4.3	79	30	0.4	0.2	235	85	90	91	
9	8800	93.0	2.0	12.8	235,000	86	1150	32	1.1	135	4.5	42	41	–	–	78	86	91	92	Expired
	10,500	90.0	6.0	11.2	325,000	53	951	40	1.4	138	3.8	43	51	–	–	35	89	91	93	
10	9000	74.0	12.0	9.0	199,000	105	–	72	3.0	135	4.0	54	14	27.0	1.6	227	88	91	97	Improved
	6600	77.0	13.0	8.8	241,000	100	–	62	3.7	132	4.5	51	11	25.0	1.5	48	92	95	97

**Table 3:** Comparison of laboratory measures between the two improved and non-improved groups undergoing the study intervention

Variable	Improved patients (***N*** = 6)	Non-improved patients (***N*** = 4)	***p***-value (t-test)
Mean age (year)	55.33 ± 21.38	60.25 ± 14.10	0.699
Baseline white blood cell count (× 10^9^/L)	8900.00 ± 3204.99	15,225.00 ± 7125.71	0.088
Baseline percentage of plasma lymphocytes (%)	8.90 ± 3.58	3.48 ± 1.96	0.038
Serum hemoglobin (mg/dL)	11.05 ± 1.69	11.30 ± 1.80	0.829
Serum platelet count (× 10^9^/L)	241,833.33 ± 139,053.11	236,250.00 ± 78,415.03	0.944
Serum c-reactive protein (mg/L)	152.00 ± 100.07	110.00 ± 56.93	0.538
Serum lactate dehydrogenase (U/L)	819.67 ± 116.17	1400.00 ± 353.55	0.066
Blood urea nitrogen (mg/dL)	30.50 ± 22.16	51.50 ± 36.35	0.284
Serum creatinine (mg/dL)	1.23 ± 0.88	1.90 ± 1.65	0.425
Serum aspartate aminotransferase (SGOT) (U/L)	68.00 ± 26.00	41.75 ± 13.91	0.141
Serum alanine aminotransferase (SGPT) (U/L)	52.00 ± 19.97	28.25 ± 8.96	0.083
Serum IL-6 (ng/mL)	122.83 ± 76.49	165.00 ± 149.29	0.568^*^

*Mann-Whitney test

**Fig. 2:**
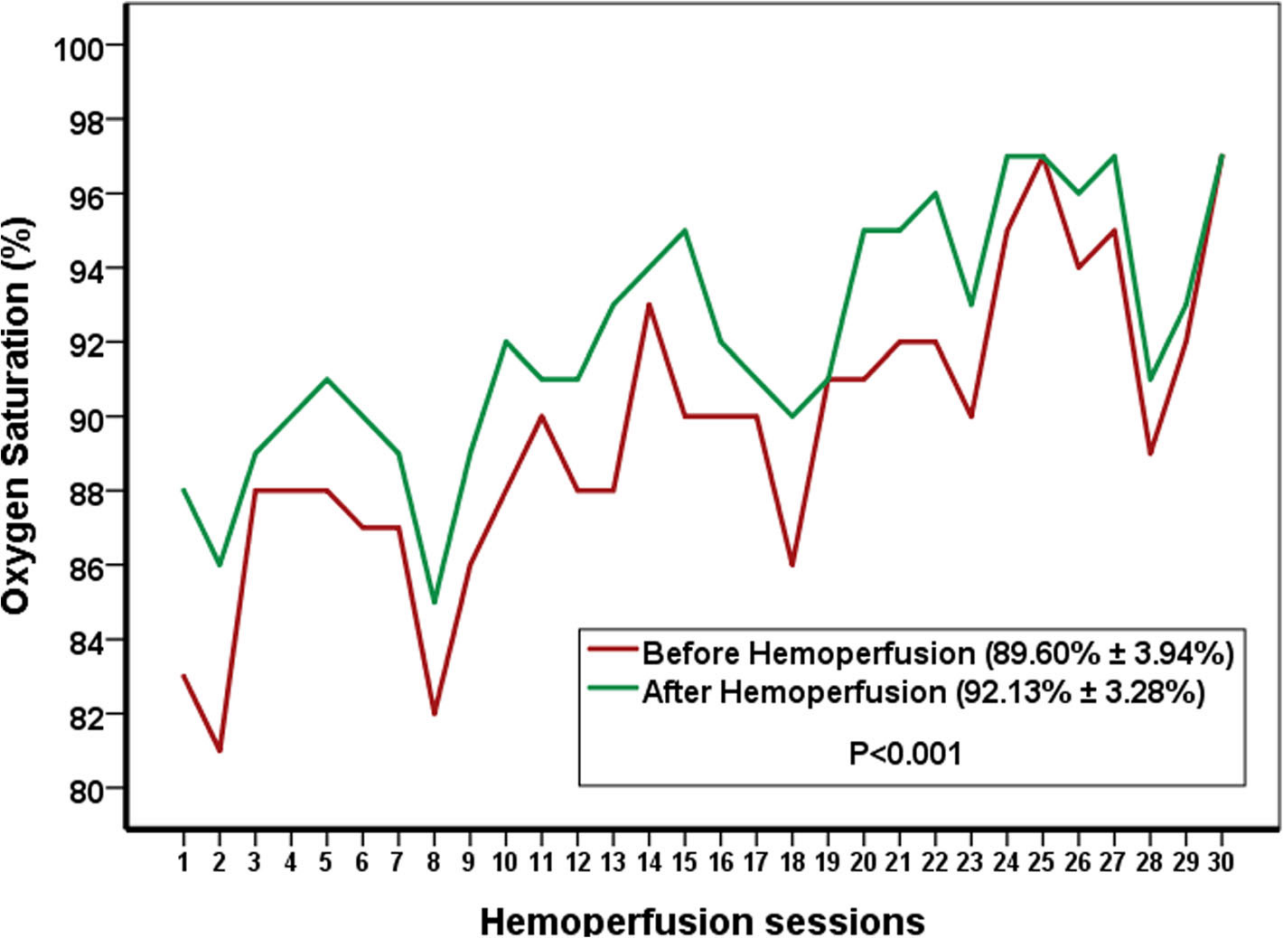
Peripheral capillary oxygen saturations before and after thirty sessions of hemoperfusion in ten patients

Peripheral capillary oxygen saturations before and after each session of hemoperfusion have been showed in Fig. 2. Mean SpO2 value was 89.60 ± 3.94% before the three sessions of intervention and increased to 92.13 ± 3.28% after the hemoperfusion (*p* < 0.001). The change of SpO2 had no significant difference in patients receiving different modes of oxygen therapy (*p* = 0.313).

## Discussion

Our results showed that three sessions of extracorporeal hemoadsorption could improve the peripheral capillary oxygen saturation in six of the ten critically ill patients with COVID-19 disease. Mean SpO2 showed a significant improvement after the intervention.

Different aspects have been listed as the potential mechanisms of organ damage and disease severity in COVID-19 patients. One of the most important mechanisms is cytokine release syndrome (also known as cytokine storm) [17–19]; and IL-6 has been represented as the most important causative cytokine in cytokine storm [10, 19]. The COVID-19 disease progresses rapidly when the cytokine storm occurs and immune responses increase [17]. Extracorporeal blood purification has been proposed as one of the treatment approaches to remove these inflammatory cytokines and could potentially be beneficial in patients with severe corona virus disease [7, 9–11]. It seems that the improvement of peripheral capillary oxygen saturation during the blood purification is related to cytokine clearance rather than reduction in volume load; because during the CRRT procedure we adjusted the fluid outflow from the patient as the same amount of the fluid input. Cytokine removal following extracorporeal therapies could prevent cytokine-induced organ damages [10]; and patients who underwent these therapeutic approaches in early phase of cytokine storm could have better clinical outcomes [11].

Interleukin-6 has been presented as a potential marker of disease severity in coronavirus infected patients. The upper limit point of serum IL-6 level in COVID-19 patients who had no severe pneumonia was reported 24.3 pg/mL, and the increased expression of IL-6 in serum is expected to predict the severity of the COVID-19 pneumonia and a poor prognosis of patients [20]. C-reactive protein (CRP) is a biomarker which may increase at early stages of coronavirus disease, and higher value of this marker can be associated with more severe pulmonary lesions in these patients [21]. In our research CRP showed a significant reduction after the intervention; and serum IL-6 decreased, although this reduction was not statistically significant. In addition to, in this study, the patients who expired showed a lower plasma lymphocytes count. Some previous studies demonstrated that COVID-19 patients with severe disease might have lower lymphocyte count compared to the mild ones [22, 23]. As we mentioned above, extracorporeal blood purification treatment can effectively remove IL-6, IL-8, IL-1β, TNF-α and so on [13], however, due to the expenditures associated with measuring various inflammatory cytokines as well as the unavailability of some laboratory kits in initial and peak periods of COVID-19 epidemic in Iran, and considering the interleukin 6 as one of the most important inflammatory cytokines, only this cytokine has been measured in this research.

In this study half of the six patients who had a chronic underlying disorder improved after the intervention. In a research in China in which 1590 laboratory-confirmed hospitalized patients with COVID-19 were evaluated about the comorbidities, hypertension and diabetes mellitus have been reported as the most prevalent comorbidities; and nearly 8% of the individuals had two or more underlying disorders [22]. A systematic review and meta-analysis reported hypertension, cardiovascular diseases, diabetes mellitus, smoking, chronic obstructive pulmonary disease, malignancy, and chronic kidney disease as the most prevalent underlying diseases among hospitalized COVID-19 patients [24]. These risk factors might compromise and deteriorate the patients’ clinical outcome. Based on our results, it seems that hemoperfusion can be beneficial in management of fluid overload, metabolic disorder, and cardiovascular dysfunction, besides to reduction of inflammatory mediators; as was mentioned in previous studies [25].

Cartridges which are used in hemoperfusion process are divided in selective and non-selective types. The Jafron resin hemoperfusion cartridges are classified as non-selective group. These cartridges are different based on the pore size distribution which determines their cutoff points for adsorption of different materials, and makes them applicable for different clinical outputs; for example HA-130 was used for improvement of uremic symptoms in chronic hemodialysis and HA-330 was effective on modulation of severe inflammatory processes [12]. We used HA-280 and HA-230 cartridges because we did not have access to other types like cytosorb, Jafron HA-380, and HA-330 in our country. There is a limited scientific evidence about the application of HA-280 and HA-230 cartridges in clinical settings [26, 27]. Previous study in which the efficacy of HA-330 resin-directed hemoperfusion has been assessed in acute respiratory distress syndrome, some considerable treatment outcomes including improved oxygenation, reduction in lung edema and histopathological signs of acute respiratory distress syndrome, and reduced circulating and alveolar cytokine levels have been resulted; and the authors concluded that this cartridge could beneficially influence the course of acute respiratory distress syndrome by attenuating systemic and pulmonary inflammatory cytokines [12]. As the cartridges we used in the process of CRRT can act as non-selective ones to absorb inflammatory cytokines such as IL-6, by conducting hemoperfusion with a mode of CVVH, more cytokines are expected to be absorbed compared to CVVH only.

Randomized trial data about the effectiveness of hemoperfusion in COVID-19 patients is lacking, however, evidence shows that this therapeutic approach is tolerable to most patients if conducted with the assistance of nephrology specialists, in order to minimize risks of infection and bleeding [11].

The strength points of this study were well-defined condition of the patients and team-working of a multidisciplinary group of experts to conduct the intervention. The most important limitations of this research were absence of a control group, small sample size of the study population, and not to present fluid balance measures in patients. Some evidence recommends PaO2/FIO2 (P/F), rather than oxygen saturation, as the best marker of oxygenation in patients with acute respiratory distress syndrome [28]; since this study was performed during the first weeks of the onset of COVID-19 epidemic, when a large number of patients were hospitalized in the state hospital, this variable was not measured.

In addition to, it is recommended to compare the impact of hemoperfusion plus CRRT on the removal of inflammatory cytokines with the effect of hemoperfusion alone in future studies. Large prospective multicenter trials in carefully selected patients are needed to definitely evaluate the efficacy of hemoadsorption in COVID-19 patients.

## Conclusion

Three sessions of extracorporeal resin-directed hemoadsorption could improve the peripheral capillary oxygen saturation in six of the ten patients with severe COVID-19 disease. Serum level of interleukin-6, as the secondary outcome of the study, decreased after the intervention, although this reduction was not statistically significant.

## Data Availability

Data supporting the results reported in the article can be found by academic researches via sending an email to the corresponding author at **dr.mouodi@gmail.com****.**

## Abbreviations

COVID-19: Corona virus disease 2019
IL-6: Interleukin-6
SpO2: Peripheral capillary oxygen saturation
PaO2: Partial pressure of oxygen in alveoli
CRP: C-reactive protein
